# Discharge Outcomes of Severely Sick Patients Hospitalized with Multidrug-Resistant Tuberculosis, Comorbidities, and Serious Adverse Events in Kyrgyz Republic, 2020–2022

**DOI:** 10.3390/tropicalmed8070338

**Published:** 2023-06-25

**Authors:** Gulzat Alumkulova, Anna Hazoyan, Elena Zhdanova, Yuliia Kuznetsova, Jaya Prasad Tripathy, Aelita Sargsyan, Olga Goncharova, Meder Kadyrov, Kylychbek Istamov, Nimer Ortuño-Gutiérrez

**Affiliations:** 1 National Center for Tuberculosis, Bishkek 720020, Kyrgyzstan; elenazhdanova201@gmail.com (E.Z.); goncharova.ncph@gmail.com (O.G.); mederkadyrow@gmail.com (M.K.); 2 Tuberculosis Research and Prevention Centre (TBRPC), Yerevan 0014, Armenia; anna.hazoyan@meduni.am (A.H.); sargsyan.aelita@gmail.com (A.S.); 3 Internal Medicine (Gastroenterology and Hepatology) Department, Yerevan State Medical University, Yerevan 0025, Armenia; 4 Alliance for Public Health, 01601 Kyiv, Ukraine; kuznetsova@aph.org.ua; 5 All India Institute of Medical Sciences, Nagpur 441108, India; ijaydoc@gmail.com; 6 School of Medicine, Osh State University, Osh City 723500, Kyrgyzstan; istamovk@gmail.com; 7 Damien Foundation, 1081 Brussels, Belgium; nimer.ortunogutierrez@damiaanactie.be

**Keywords:** SORT-IT, patient discharge, HIV infections, tuberculosis, multidrug-resistant, diabetes mellitus, malnutrition, anemia, Kyrgyzstan, electrolytes

## Abstract

Patients with multidrug-resistant tuberculosis (MDR-TB) who have comorbidities, complications, and experience serious adverse events (SAEs) are at substantial risk of having unfavorable hospital outcomes. We assessed characteristics and discharge outcomes of 138 MDR-TB patients hospitalized in the National Referral Center of Bishkek, Kyrgyz Republic, from January 2020 to August 2022. The main clinical characteristics included pulmonary complications (23%), malnutrition (33%), severe anemia (17%), diabetes mellitus (13%), viral hepatitis B and C (5%), and HIV infection (3%). Of those patients, 95% were successfully managed and discharged from hospital. Seven patients had unfavorable discharge outcomes (three patients died and four had a worsened clinical condition). Comorbidities (diabetes, and/or HIV), severe anemia, pulmonary complications, cardiovascular disorders, alcohol abuse, and SAEs were associated with unfavorable discharge outcomes. Sixty-five percent of the patients had SAEs, with electrolyte imbalance (25%), gastrointestinal disease (18%), hepatotoxicity (16%), and anemia (14%) being the most frequent. Successful resolution occurred in 91% of patients with SAEs. In summary, our study documented that sick patients who were hospitalized with MDR-TB were well managed and had good hospital discharge outcomes, despite the fact that they had comorbidities, complications, and SAEs. This information should assist in the referral and management of such patients in the future.

## 1. Introduction

Multidrug-resistant tuberculosis (MDR-TB) is caused by bacteria that are resistant to rifampicin and isoniazid, the most powerful first-line drugs for the treatment of tuberculosis [1]. In 2021, the estimated number of new MDR-TB new cases was 450,000 globally, representing a 3.1% increase compared to 2020 as a consequence of the COVID-19 pandemic. Globally, in 2021 the treatment success rate of MDR-TB patients was poor and only reached 60% [2]. Favorable treatment outcomes depend mainly on an adequate treatment regimen combination of bactericidal and sterilizing anti-tuberculosis drugs, and the duration and safety of the treatment regimen. The World Health Organization (WHO) currently recommends a 6-month regimen based on new drugs such as bedaquiline, pretomanid, linezolid, and/or moxifloxacin (BPaL, BPaLM) for the treatment of MDR-TB [3], replacing a former recommendation of a shorter treatment regimen (STR) of 9-12 months. Both BPaLM and STR had treatment success rates above 80% compared to 50% for the 18-month former long-duration treatment regimens [4,5,6,7]. The timely identification and proper management of complications of MDR-TB, associated comorbidities (e.g., HIV, diabetes mellitus, viral hepatitis) and serious adverse events also influence treatment outcomes [8]. Therefore, a decentralized model of care [9], where severely ill MDR-TB patients faced with life-threatening conditions who can be referred in a timely way to specialized centers has been shown to work and is associated with better treatment success [1,10].

The Kyrgyz Republic is one of 30 countries worldwide with the highest MDR-TB burden, with an estimated incidence of 49 MDR-TB patients per 100,000 population, and 917 notified cases in 2021 [11]. All MDR-TB patients with complications or comorbidities and/or serious adverse events (SAEs) are referred to the National TB Center in Bishkek for specialized management.

The National Tuberculosis Program (NTP) in Kyrgyz Republic has been adopting the WHO recommendations for MDR-TB care and treatment regimens, including the recent recommendation of the 6-month BPaL/BPaLM regimen [12]. In 2017, a study conducted in Kyrgyz Republic showed that the STR for MDR-TB achieved a treatment success of 83% compared to 50% with the conventional 18-month-long treatment regimen [13]. However, the treatment success rate reported in 2022 among all MDR-TB patients (including ambulatory and hospitalized patients) was 72% compared to 82% in drug-susceptible TB [11]. The MDR-TB treatment success rate is probably affected by the treatment outcome of hospitalized MDR-TB cases who are those most severely ill. Hospitalized MDR-TB patients represent a very ill cohort as treatment regimens use last-resort drugs for complex patients affected by comorbidities, complications, and serious adverse events. To date, there has been no formal assessment of the management of hospitalized MDR-TB cases in Kyrgyz Republic. Studying their management and treatment as well as their hospital discharge outcomes will inform and guide the NTP in their quest to improve coverage of the quality of care in specialized referral services and will allow the NTP to therefore adhere to the principle of “leaving no one behind”.

Our study aimed to assess the effectiveness of the management of patients with MDR-TB who were hospitalized in Kyrgyz Republic. Our specific objectives were to document in patients hospitalized with MDR-TB at the National Center for Tuberculosis in Bishkek from January 2020 to August 2022: (1) their sociodemographic and clinical characteristics; (2) the type of SAEs and their associated factors; and (3) their hospital discharge outcomes and their associated socio-demographic and clinical factors.

## 2. Materials and Methods

### 2.1. Study Design

This was a cohort study using secondary data from the NTP.

### 2.2. Study Setting

The Kyrgyz Republic is located in Central Asia and has a population of approximately 6.5 million and is ranked as a medium human development country at 118 among 191 countries [14].

#### 2.2.1. Diagnosis and Treatment of MDR/RR-TB

The National Tuberculosis Program (NTP) initiated the programmatic management of drug-resistant TB (PMDT) in 2005. Patients with presumptive MDR-TB are detected at the primary health care level where sputum samples are transported to 24 GeneXpert diagnosis centers and referred to 1 of 61 TB cabinets for ambulatory treatment after confirmation. Diagnosis of MDR-TB is based on phenotypic drug-susceptibility testing (DST) from liquid and solid culture media and molecular tests such as line probe assays (LPA)-GenoTypeMTBDRplus for the detection of rifampicin and isoniazid resistance, and GenoTypeMTBDRsl for the detection of resistance to fluoroquinolones (FQ) and second-line injectables (SLI) performed at the National Reference Laboratory (NRL).

Once MDR-TB is confirmed, patients are screened for comorbidities before the start of TB treatment. The medical Concilium (expert commission including MDR-TB experts) in each of the seven regions conducts a patient assessment, confirms or corrects the diagnosis, recommends the optimal treatment regimen, and determines and or confirms the treatment outcomes. The Central Concilium of the National Center for Tuberculosis makes the final decisions on controversial issues and monitors the fulfillment of duties by regional Concilium. Four treatment regimens were recommended during the study period (see Table 1).

#### 2.2.2. The Referral System

The National TB Referral Center admits patients with (i) bacteriologically confirmed pulmonary TB with MDR/RR-TB, (ii) associated clinically diagnosed comorbidities, (iii) severe and moderate adverse drug events, and (iv) indications for thoracic surgery. The TB referral center has two dedicated TB specialist physicians, fifteen nurses, and nursing aids for inpatient care. The TB Referral Center has 50 beds, including 2 in the intensive care unit. Treatment includes the provision of medical ancillary drugs, fluids, nutritional support adapted to the nutritional status and comorbidities, and oxygen. All medical care related to TB is provided free of charge. Clinical monitoring is conducted daily during the first weeks in the event of hospitalization and at least every week during outpatient treatment until the patient reaches a stable level at the peripheral level (polyclinics). After stabilization, the patient is managed at the peripheral level and if a new complication arises the patient will be referred to the hospital again.

#### 2.2.3. Follow-Up of Patients under Treatment

Patients are monitored clinically and bacteriologically (i.e., weight, sputum microscopy, and culture) on a monthly basis. Additional tests are carried out according to the TB treatment regimens. These may include: monthly audiometry if patients receive second-line injectable drugs; electrocardiogram readings (ECG) at the 2nd, 4th, 8th, 12th, and 24th week after starting to take bedaquiline or delamanid-monthly ECG is performed if the patients are taking other drugs besides bedaquiline such as fluoroquinolones and clofazamine; monthly assessments by an ophthalmologist if patients take linezolid; and monthly whole full blood count, renal and liver function tests.

If the SAEs in our study were Grade 3 (severe but not life-threatening), their management included clear restriction of activities and medical care that included hospitalization or prolongation of hospitalization if necessary. If the SAEs were Grade 4 (profound and life threatening), their management included tight restriction of activities, significant urgent medical care, and hospitalization [12].

### 2.3. Study Population and Study Period

All MDR/RR-TB patients admitted and managed at the Pulmonary Tuberculosis Department of the National TB Center in Bishkek from January 2020 to August 2022.

### 2.4. Recording and Reporting System

The NTP recommends the use of standardized tools for management and reporting such as TB patient cards, TB registers, Laboratory TB registers, and quarterly and yearly reports. Since 2018, an e-Health system that includes data in electronic format with NTP tools and that links all health facilities from the peripheral to the referral center was introduced. The e-Health data are cross-checked with paper-based forms and validated during supervision visits by the NTP, the Mandatory Health Insurance Fund, and partners such as the United Nations Development Programme (UNDP), and these visits are conducted at least once a year.

### 2.5. Data Collection and Analysis

The data variables were extracted by the responsible internet technology officer and the Principal Investigator from digital e-Health software at the National Center for Tuberculosis in Bishkek. The data were cross-checked with paper-based records at the hospital and the peripheral level: i.e., TB registers by the principal investigator and the officer responsible for MDR-TB care at the peripheral level. The data collection and verification were carried out between January and April 2023. Analysis was conducted using Stata software (v12, StataCorp, College Station, TX, USA). Demographic and clinical profiles of study participants included frequencies and proportions (for categorical variables) and mean (and standard deviation) or median (and interquartile range) for continuous variables as appropriate. We described the frequency of the sociodemographic and clinical characteristics of patients hospitalized with the region of residence of referrals and factors associated with safety and effectiveness (i.e., comorbidities, complications, SAEs, and type of treatment regimen). Treatment outcomes of hospitalized patients were described using frequencies and proportions classified into successful (discharged and referred to the peripheral level or referred to the regional hospitals) and unsuccessful outcomes (worsened clinical condition or death) in association with the type of treatment regimen. Multivariate analysis was not performed as the unfavorable outcomes came to small numbers.

## 3. Results

During January 2020 and August 2022, we enrolled into our study 138 (54%) of 243 patients who were hospitalized with MDR/RR-TB. Reasons for not enrolling the other 105 patients were missing data on sociodemographic variables, patients affected by drug-susceptible TB without complications, and those with extensively drug-resistant TB (XDR-TB).

### 3.1. Sociodemographic Characteristics

Sociodemographic and clinical characteristics are shown in Table 2. Of the 138 MDR/RR-TB patients, 69(50%) were aged 18–35 years old, most patients were male (57.2%), more than 70% came from the Chui region and Bishkek city, and the majority of the patients 125(90%) were unemployed.

### 3.2. Clinical Characteristics

Of the 138 patients with MDR/RR-TB, 63% were classified as having new TB. There were 101 (73%) who had only pulmonary disease and 27% who had both pulmonary and extra-pulmonary disease. There were 105 (76%) patients who were smear-positive on light microscopy and 24% had mycobacterial culture and/or molecular tests showing drug resistance. According to the drug-resistance profile, all patients had MDR/RR-TB, with 20(14%) having pre-XDR-TB (12 resistant to fluoroquinolones and 8 resistant to second-line injectable drugs).

There were 50 patients with a body mass index (BMI) showing malnutrition (33%) and 4% were overweight. There were 32 (23%) with pulmonary complications. Among those with comorbidities, 17% had severe anemia, 13% had diabetes mellitus, 5% had viral hepatitis (B and C), and 3% were infected with HIV. A total of 66% of patients were being treated with an individualized longer regimen. The median duration of hospitalization was 59 days, with an interquartile range ranging from 32 to 88 days.

### 3.3. Treatment Outcomes of MDR-TB Hospitalized Patients

There were 7 (5%) patients with unfavorable outcomes (these included a worsened clinical condition or death). There were 131 patients (95%) with a favorable outcome who were discharged and referred for continuation of treatment to the peripheral level or the regional hospitals (see Table 3).

Among the seven MDR-TB patients with unfavorable outcomes, one patient who worsened was treated with an oral shorter regimen, and six were treated with individualized longer regimens (with three having a worsened clinical condition and three dying). Among those who worsened, one had a cholecystectomy, two had alcohol consumption disease, and one had pulmonary complications. Regarding sociodemographic characteristics of these seven patients, 57% were aged 18–35 years, 57% were male, all were unemployed, and 29% were smokers. Regarding TB clinical characteristics, 43% had pulmonary and extrapulmonary disease, 57% had malnutrition, 43% had relapsed TB, 86% were sputum smear-positive, and all had MDR-TB. Other clinical manifestations included severe anemia (42%), cardiovascular disorders (42%), pulmonary complications (29%), diabetes mellitus (14%), HIV (29%), and SAEs (43%). The median duration of hospitalization of the seven patients was 4 days with an interquartile range from 3 to 12 days.

### 3.4. Serious Adverse Events among MDR-TB Hospitalized Patients

There were 88 (65%) patients with SAEs, with a total of 141 episodes (as some patients had more than one concurrent SAEs). Among Pre-XDR there were 13/20 (65%) with SAEs. The main SAEs were electrolyte imbalance (25%), gastrointestinal side effects (18%), hepatotoxicity (16%), and anemia (14%). There were no events related to skin pigmentation, phlebitis, loss of vision, neuropathy, and ototoxicity (see Table 4).

SAEs occurred in all age categories (with a frequency > 60%) with the highest frequency observed in those aged 56–66 years (73%). Of 79 males, 65% had SAEs. All the regions were home to patients with SAEs except Batken. Sixty-five percent of patients with MDR-TB and only pulmonary disease had SAEs. Among the different population groups, those more affected with SAEs were: employed (77%), homeless (75%), and smokers (58%). Among the comorbidities, the majority of SAEs were associated with viral hepatitis (100%), severe anemia (89%) and HIV infection (75%). More than 77% of patients with SAEs were hospitalized for more than 30 days. The oral shorter treatment regimen had the highest proportion of SAEs (69%). The statistically significant factors associated with the occurrence of SAEs were severe anemia (RR 1.5, 95% CI 1.2–1.9) and longer duration of hospitalization (>31 days) (RR 6.9, 95% CI 1.1–44.3). The frequency of risk factors associated with SAEs are shown in Table 5.

## 4. Discussion

Our study is the first in the Republic of Kyrgyz to document the unfavorable outcomes at the discharge of patients hospitalized with MDR-TB with associated comorbidities, complications, and SAEs. Among 138 MDR-TB patients hospitalized, only seven (5%) had unfavorable outcomes (death or a worsened clinical condition). This proportion is lower compared to a study that included drug-susceptible and drug-resistant TB patients admitted to a tertiary hospital in India (14%) [15].

All patients with unfavorable outcomes at hospital discharge had at least one comorbidity (diabetes and HIV) associated with other clinical conditions such as severe anemia, pulmonary complications, cardiovascular disorders, alcohol abuse, and more than 40% also had SAEs. Alcohol abuse is elsewhere associated with a statistically significant higher risk of developing SAEs in hospitalized patients [16], but also with a higher risk of loss to follow-up after discharge from hospital [17].

The median duration of the hospitalization in those with unfavorable outcomes was 4 days (IQR 3–12) compared to 59 days (IQR 32–87) among the total MDR-TB patients enrolled in our study. This illustrates their advanced MDR-TB disease and the delay of referral of ill patients for proper management which might have increased their chances of survival. In a study conducted in South Africa, another high MDR-TB burden setting, the median number of days of hospitalizations was 399 which was mainly explained by the high prevalence of HIV infection among their MDR-TB patients [18].

In our study, 65% of the hospitalized patients had SAEs, showing the relevance of referral for specialized management at the referral center. In total, 77% of patients with SAEs were hospitalized for more than 30 days, illustrating that long stay is required for the proper management of SAEs. The most frequent SAEs included electrolyte imbalance, gastrointestinal disorders, hepatotoxicity, and anemia. These proportions are higher compared to the SAEs found in a study involving two referral hospitals in Uganda where the problems were mainly gastrointestinal disorders and arthralgia. Other SAEs reported in the same study in Uganda were ototoxicity and vision change [16], which were not experienced in our study. Ototoxicity is explained by the large number of patients treated with regimens containing second-line injectables in the two referral hospitals in Uganda [16].

The frequency of SAEs in our study was associated with 62% of patients treated with individual longer treatment regimens, with a relative risk that was not statistically significant. In the study in the two referral hospitals in Uganda, there was a statistically significant association between developing SAEs and the longer standardized regimens (24 months) and individualized regimens [16]. Severe anemia was significantly associated with SAEs, which might be related to the use of linezolid as observed in regimens that include this drug [19].

There are a number of important implications of our study that might be useful for improving the hospital care of patients with MDR-TB admitted to the referral National TB Center in Bishkek.

First, the low rate of unfavorable outcomes at discharge of hospitalized patients with MDR-TB highlights the expertise required to treat those referred with the new BPaL/M regimen which is associated with other important SAEs. The pivotal Nix-TB study that recommended BPaL reported that neuropathy, myelosuppression, and anemia were associated with linezolid [19].

Second, access to early diagnosis can be improved by ensuring the availability of more sensitive automated molecular technology for the diagnosis of MDR-TB such as Xpert MTB/XDR [20]. Furthermore, a rapid turnaround of phenotypic drug susceptibility tests from liquid culture is needed in case of suspected resistance to new drugs such as bedaquiline, linezolid, and pretomanid.

Third, the referral system should be strengthened to avoid delays, to monitor multiple episodes of hospitalizations, and to improve the hospital outcomes of patients managed at the National TB Center in Bishkek so that they can be managed at the peripheral or intermediate level for the continuation of treatment. As the most frequent SAEs were electrolyte imbalance, hepatotoxicity, gastrointestinal disorders and anemia, the active tuberculosis drug-safety monitoring, and management (aDSM) should be reinforced at the peripheral level in order to reduce the frequency of these unfavorable outcomes.

Fourth, assessing adherence to treatment during hospital admissions must be incorporated as an essential indicator of quality of care. As MDR-TB treatment regimens incorporate last-resort drugs, adherence to anti-TB treatment is essential despite the presence of comorbidities, complications, and SAEs. A study in South Africa showed that MDR-TB patients treated with amikacin, PAS, and ethionamide had lower adherence to treatment during hospitalization [18]. These drugs are classified under Group C of WHO classification of second-line drugs and are used in individualized treatments [21].

Finally, the recording and reporting system must be strengthened to ensure an adequate and timely flow of data between the different levels of the health system, but at the same time it needs to ensure completeness of key indicators for the management of hospitalized MDR-TB patients.

Our study had several strengths. The data of hospitalized MDR-TB patients was cross-checked with paper-based records and validated by the medical staff involved in patient care and by the principal investigator. We also followed the STROBE guidelines in the conduct and reporting of this observational study [22]. Our study was also included as a priority research topic by the NTP to improve the quality of care and treatment success for MDR-TB patients enrolled in the new BPaL/M regimen at the National TB Referral Center in Bishkek.

The main limitation of our study is that we could not enroll all hospitalized patients because of missing data. We could also not track patients during the post-discharge period until the end of the treatment regimen. A study in South Africa found that there is a higher risk of stopping treatment after the first two months of hospital discharge, with alcohol consumption being the main risk factor [17]. Finally, we could not assess if the patients had multiple hospitalizations. A study in South Africa documented that more than 50% of their patients had multiple episodes of hospitalization [18].

## 5. Conclusions

In the National TB Referral Center in Bishkek, 65% of the hospitalized MDR-TB patients had SAEs. The proportion of MDR-TB hospitalized that died and worsened was low, illustrating adequate management at the TB Referral Center. Our study identified potential action points that, if taken up, could improve the quality of care, in particular, identifying those at higher risk of unfavorable outcomes and addressing specific areas of the referral system.

## Figures and Tables

**Table 1:** Type of multidrug-resistant TB regimens in Kyrgyzstan, 2020–2022.

Regimen, Drugs, and Duration of Treatment	Indications
STR with injectables 4–6 (Am-Lfx or Mfx-Cfz-E-H^HD^-Pto/5 (Mfx-Cfz-E-Z)	(1) MDR/RR-TB susceptible to drugs included (except H), and not treated for ≥one month; (2) Non-disseminated form of pulmonary TB; (3) Absence of contact with patients with known resistance to second-line drugs; (4) No concomitant pulmonary and extra-pulmonary TB; (5) Extra-pulmonary TB, except nervous system, spondylitis, or any extrapulmonary and HIV; (6) No pregnancy; (7) Absence of allergy to the drugs; (8) No intolerance to any drug or no high risk of drug toxicity and/or interactions; and (9) Antiretroviral drugs by patients with HIV infection.
STR all-oral 4–6 (Bdq-Lfx or Mfx-Cfz-E-H-(HD)-Pto/5 (Mfx-Cfz-E-Z)	
BPaL/M * 6 (Bdq, Pa, Lzd/Mxf)	
Individual longer regimens Different drug combinations for 18–20 months according to the WHO recommendations including a combination of the following drugs: Bdq, Cm, Km, Am, Mfx, Lzd, Cfz, Lfx, Cs, Dlm, Z, etc.	Whenever STR and BpaL/M indications are not met

STR = Shorter treatment regimen; Am = amikacin; Mfx = moxifloxacin or Lfx = levofloxacin, Pto = prothionamide, Cfz = clofazimine, Z = pyrazinamide, H^HD^ = isoniazid at high dose, and E = ethambutol (E), Z = pyrazinamide, Bedaquiline = Bdq, P = Pretomanid, Cm = capreomycin, Km = kanamycin, Cs = cycloserine, Dlm = delamanid; * Moxifloxacin to be added only if fluoroquinolone susceptibility; WHO = World Health Organization.

**Table 2:** Sociodemographic and clinical characteristics of MDR-TB patients referred and admitted to the National Center for Tuberculosis in Bishkek, Kyrgyzstan, from 2020 to 2022.

Characteristics	N	(%)
Total	138	(100)
Sociodemographic		
Age groups (in years)		
0–17	5	(3.6)
18–35	69	(50)
36–55	40	(28.9)
56–65	15	(10.9)
>66	9	(6.5)
Gender		
Male	79	(57.2)
Female	59	(42.8)
Region		
Bishkek city	53	(38.4)
Osh	4	(2.9)
Batken	2	(1.4)
Jalalabad	10	(7.2)
Issyk-Kul	9	(6.5)
Naryn	3	(2.2)
Talas	3	(2.2)
Chui	54	(39.1)
Population groups *		
Employed	13	(9.4)
Homeless	4	(2.9)
Smokers (self-reported)	26	(18.8)
Clinical characteristics		
Pulmonary and extrapulmonary involvement		
Yes	37	(26.8)
No	101	(73.2)
Body Mass Index		
Undernutrition	45	(32.6)
Overweight	5	(3.6)
Normal	88	(63.7)
Type of TB		
New	86	(62.3)
Relapse	52	(37.7)
Smear status at diagnosis		
Positive	105	(76.1)
Negative with molecular test showing resistance and/or culture positive	33	(23.9)
Drug resistance		
MDR/RR	138	(100)
Pre-XDR/Extensive drug resistant	20	(14.5)
Pulmonary complications	32	(23.2)
Other Clinical manifestations *		
Severe anemia	23	(16.7)
Cardiovascular disorders	14	(10.1)
Diabetes mellitus	18	(13.0)
Hepatitis B	6	(4.4)
Hepatitis C	1	(0.7)
HIV	4	(2.9)
Duration of hospitalization (median/IQR)	59	(32–88)
Type of regimen		
Oral shorter regimen	45	(32.6)
Injectable shorter regimen	2	(1.5)
Individual longer treatment regimen	91	(65.9)

TB: tuberculosis; MDR/RR: Multidrug- and rifampicin-resistant TB; XDR: Extensively Drug Resistant; IQR: Inter Quartile Range; HIV: Human Immunodeficiency Virus; * patients might belong to >1 group.

**Table 3:** Treatment outcomes of MDR-TB patients referred and admitted to the National Center for Tuberculosis in Bishkek, Kyrgyzstan, from 2020 to 2022 stratified by the type of regimen.

	Oral Shorter		Injectable Shorter Regimen		Individual Treatment Regimen	
	*n*	(%)	*n*	(%)	*n*	(%)
Total	45	(100)	2	(100)	91	(100)
Favorable hospital discharge outcomes						
Referral to regional hospital	10	(22.2)	0	(0)	27	(29.7)
Referral to peripheral level	34	(75.6)	2	(100)	58	(63.7)
Unfavorable hospital discharge outcomes						
Died	0	(0)	0	(0)	3	(3.3)
Worsened	1	(2.2)	0	(0)	3	(3.3)

MDR-TB: Multi-Drug Resistant Tuberculosis.

**Table 4:** Frequency and outcomes of Serious Adverse Events during hospitalization among MDR-TB patients referred and admitted to the National Center for Tuberculosis in Bishkek, Kyrgyzstan, from 2020 to 2022.

Serious Adverse Events (SAEs)	Frequency of SAEs *n* (%)	Resolved *n* (%)
Total	141 (100)	129 (91)
Electrolyte imbalance	35 (25.4)	34 (97)
Gastrointestinal	25 (18.1)	23 (92)
Hepatotoxicity	22 (15.9)	21 (95)
Anemia	19 (13.8)	15 (79)
Nephrotoxicity	11 (7.9)	11 (100)
Arthralgia	11 (7.9)	10 (90)
Psychiatric abnormalities	10 (7.3)	8 (80)
Cardiotoxicity	8 (5.8)	7 (88)

SAE: Serious Adverse Events; MDR-TB: Multi-Drug Resistant Tuberculosis.

**Table 5:** Risk factors associated with serious adverse events (SAEs) among MDR-TB patients hospitalized at the National Center for Tuberculosis in Bishkek, Kyrgyzstan, from 2020 to 2022.

Characteristics	Total	Occurrence of at Least One Episode of SAE	Crude RR (95% CI)	
		*n* (%)		
Total	138	88 (64.6)		
Sociodemographic				
Age groups (in years)				
0–17	5	3 (60)	Ref.	
18–35	69	44 (63.8)	1.1	(0.5–2.2)
36–56	40	24 (60)	1.0	(0.5–2.1)
56–65	15	11 (73.3)	1.2	(0.6–2.7)
>65	9	6 (66.7)	1.1	(0.5–2.6)
Gender				
Female	59	37 (62.7)	Ref.	
Male	79	51 (64.6)	1.0	(0.8–1.3)
Region				
Osh	4	3 (75)	Ref.	
Bishkek city	53	33 (62.3)	0.8	(0.5–1.5)
Batken	2	0 (0)	NA	NA
Jalalabad	10	5 (50)	0.7	(0.3–1.5)
Issyk-Kul	9	6 (66.7)	0.9	(0.4–1.8)
Naryn	3	2 (66.7)	0.9	(0.3–2.4)
Talas	3	3 (100)	NA	NA
Chui	54	36 (66.7)	0.9	(0.5–1.6)
Clinical characteristics				
Extrapulmonary involvement				
No	101	66 (65.4)	Ref.	
Yes	37	22 (59.5)	1.9	(0.7–1.2)
Body Mass Index				
Normal	88	57 (64.8)	Ref.	
Undernutrition	45	29 (64.4)	0.6	(0.2–1.8)
Overweight	5	2 (40)	0.9	(0.8–1.3)
Type of TB				
New	86	58 (67.4)	Ref.	
Relapse	52	30 (57.7)	0.8	(0.6–1.1)
Smear status at diagnosis				
Negative with culture and/or molecular drug susceptibility tests showing resistance	33	17 (51.5)	Ref.	
Positive	105	71 (67.6)	1.3	(0.9–1.9)
Population groups				
Unemployed	125	78 (62.4)	Ref.	
Employed	13	10 (77)	1.2	(0.9–1.7)
Homeless	4	3 (75)	1.2	(0.7–2.1)
Smokers	26	15 (57.7)	0.9	(0.6–1.3)
Other Clinical manifestations				
Severe anemia	19	17 (89.5)	1.5	(1.2–1.9)
Cardiovascular disorders	14	10 (71.4)	1.4	(0.8–1.6)
Pulmonary complications	22	14 (63.6)	1.0	(0.7–1.4)
Diabetes mellitus	18	12 (66.7)	1.1	(0.7–1.5)
Hepatitis B	6	6 (100)	NA	NA
Hepatitis C	1	1 (100)	NA	NA
HIV	4	3 (75)	1.2	(0.6–2.1)
Duration of hospitalization				
<7 days	9	1 (11)	Ref.	
8–30 days	23	5 (27.7)	1.9	(0.3–14.5)
>31 days	106	82 (77.3)	6.9	(1.1–44.3)
Type of regimen				
Oral shorter regimen	45	31 (68.9)	Ref.	
Injectable shorter regimen	2	0 (0)	NA	NA
Individual treatment regimen	91	57 (62.6)	0.9	(0.7–1.2)

TB: tuberculosis; MDR/RR: Multidrug- and rifampicin-resistant TB; XDR: Extremely Drug Resistant; HIV: Human Immunodeficiency Virus; RR = Risk Ratio; CI = confidence intervals; Ref. = reference; NA = Not applicable.

## Data Availability

The data included in this study are available on request from the corresponding author.
